# Design, synthesis, and biological evaluation of 6-aryl-3-(3,4,5-trimethoxyphenyl)imidazo[1,2-a]pyridine derivatives as novel tubulin inhibitors with potent anticancer efficacy

**DOI:** 10.1080/14756366.2026.2686919

**Published:** 2026-06-17

**Authors:** Wen Xu, Yujing Zhang, Qianqian Xu, Haibo Zhao, Liya Cui, Chao Wang

**Affiliations:** ^a^ Department of Pharmacy, The Affiliated Hospital of Qingdao University, Qingdao, Shandong, China; ^b^ The Affiliated Cardiovascular Hospital of Qingdao University, Qingdao University, Qingdao, Shandong, China; ^c^ Cancer Institute, The Affiliated Hospital of Qingdao University, Qingdao University, Qingdao, Shandong, China

**Keywords:** Tubulin polymerisation inhibitor, colchicine-binding site, imidazopyridine, molecular docking, antiproliferative activity

## Abstract

Tubulin, the fundamental component of microtubules, remains a critical target in anticancer therapy. The development of small-molecule tubulin polymerization inhibitors continues to drive the discovery of novel chemotherapeutic agents. Through systematic analysis of known tubulin inhibitors and in silico binding pocket models, a focused series of 6-aryl-3-(3,4,5-trimethoxyphenyl)imidazo[1,2-a]pyridine derivatives was rationally designed and synthesized. Compound 8o exhibited superior antiproliferative potency, with IC₅。 values of 0.050-0.078 µM against HeLa, HCT116, and MCF-7 cancer cell lines. Mechanistic investigations confirmed that 8o effectively inhibits tubulin polymerization, disrupts the microtubule cytoskeleton, induces G₂/M phase arrest, and triggers apoptosis. Preliminary physicochemical assessment indicated that 8o adheres to Lipinski’s rule of five, supporting favorable drug-likeness. Collectively, these findings identify 8o as a potent, drug-like colchicine-site tubulin inhibitor with compelling anticancer efficacy, meriting further investigation as a promising lead compound.

## Graphical abstract

**Figure:**
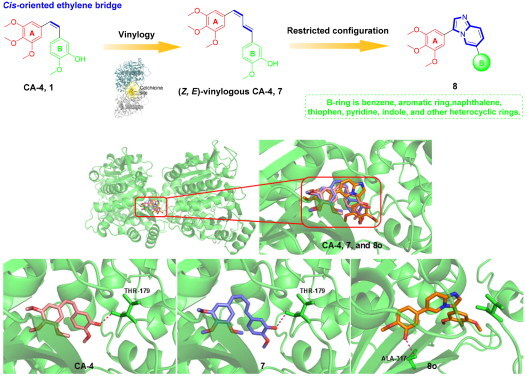

## Introduction

Microtubules, dynamic cytoskeletal polymers formed by *α*/*β*-tubulin heterodimers, govern essential cellular functions including chromosome segregation, organelle transport, and cell division^1^^,^^2^. The precise regulation of microtubule dynamics – particularly their assembly and disassembly – serves as a cornerstone of antimitotic chemotherapy, since disrupting this balance effectively halts proliferation and triggers apoptotic death in malignant cells^3^. While established microtubule-targeting agents such as taxanes and vinca alkaloids remain clinically useful, their long-term utility is often limited by multidrug resistance and adverse side effects^4^. Consequently, targeting the colchicine-binding site (CBS) on *β*-tubulin has emerged as a compelling alternative strategy, potentially circumventing common resistance mechanisms while offering distinct pharmacological profiles^5^.

**Figure 1.**
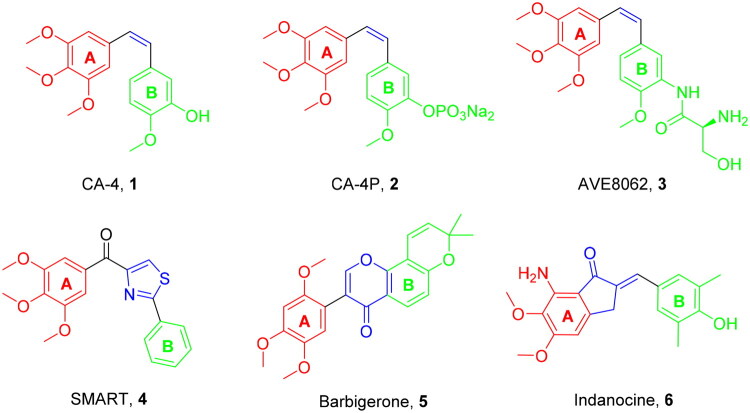
The chemical structure of the well-known CBSI.

Over the past two decades, substantial medicinal chemistry efforts have yielded a wide spectrum of colchicine-binding site inhibitors (CBSIs), with several advancing into clinical development^6^^,^^7^. Notable examples include the natural product combretastatin A-4 (**CA-4**, **1**)^8^, its phosphate prodrug fosbretabulin (**2**)^9^, ombrabulin (AVE8062, **3**)^10^, SMART (**4**)^11^, barbigerone (**5**)^12^, and indanocine (**6**)^13^ (Figure 1). Despite encouraging preclinical results, many CBSI candidates have encountered translational challenges related to suboptimal pharmacokinetics, metabolic instability, or narrow therapeutic windows^14^^,^^15^. Recent advances in the field have continued to yield novel CBSIs with improved pharmacological profiles, including structurally diverse scaffolds that address issues of metabolic stability and resistance^16–18^. Notably, several recent studies have reported innovative heterocyclic templates that successfully mimic the bioactive conformation of **CA-4** while overcoming its configurational instability, providing valuable inspiration for our scaffold-hopping strategy^19^^,^^20^. These limitations underscore the ongoing need for innovative molecular scaffolds that combine potent tubulin inhibition with improved drug-like properties.

**Figure 2.**
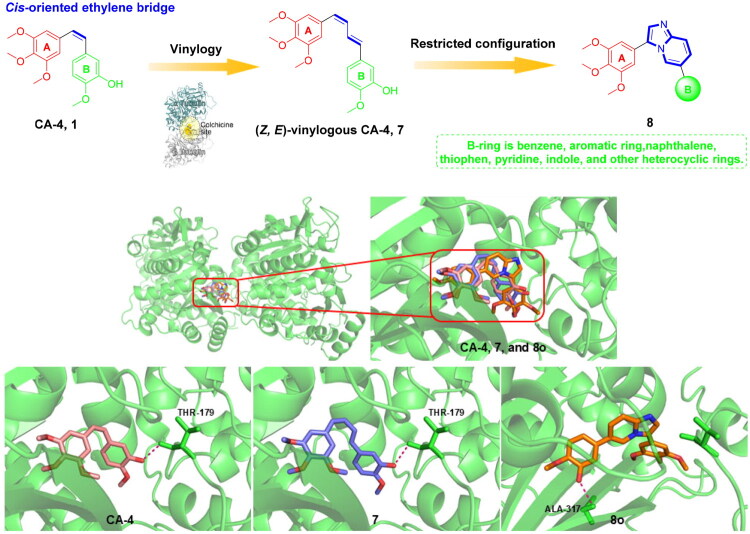
The rational design of target compounds.

A principal challenge in CBSI design stems from the configurational instability of key pharmacophores. **CA-4**, a benchmark CBSI, exerts potent antiproliferative activity *via* its *cis*-configured stilbene core, which competitively inhibits tubulin polymerization^21^. Structural modifications that preserve this bioactive geometry – such as the (*Z,E*)-butadiene linker in analogue **7** (Figure 2) – can retain potency^22^. However, the *cis*-olefin in such compounds remains prone to *in vivo* isomerisation to the thermodynamically favoured but inactive *trans*-form, substantially diminishing therapeutic efficacy^23^^,^^24^. One proven tactic to overcome this liability involves rigidification: replacing the flexible double bond with a conformationally constrained heterocycle that mimics the spatial arrangement of the aryl pharmacophores while preventing isomerization^25–27^.

From the perspective of drug design and lead-compound optimisation, our strategy was built upon a systematic pharmacophore-based approach. The 3,4,5-trimethoxyphenyl moiety, which is indispensable for anchoring within the colchicine-binding pocket through hydrophobic interactions with residues such as *β*-Lys352 and *β*-Asn349, was retained as a conserved structural motif. The key innovation lies in the bioisosteric replacement of the isomerisation-prone (*Z*,*E*)-butadiene linker of lead compound **7** with a fused, planar imidazo[1,2-a]pyridine system. This rigid heterocyclic scaffold permanently locks the relative orientation of the two aryl units in a geometry that mimics the bioactive conformation of **CA-4**, thereby circumventing the configurational liability inherent to flexible analogues. The imidazo[1,2-a]pyridine core was selected for its privileged status in medicinal chemistry, favourable physicochemical profile, and demonstrated utility in anticancer drug discovery^28^^,^^29^. Through this scaffold-hopping strategy, we aimed to obtain a novel class of structurally stabilised, potent tubulin polymerisation inhibitors with enhanced metabolic stability and promising anticancer activity.

Imidazo[1,2-a]pyridine represents a structurally versatile and pharmacologically privileged heterocycle, extensively studied for its favourable physicochemical profile and diverse bioactivities, including notable anticancer effects. Its planar, electron-rich architecture offers an attractive template for designing conformationally restricted bioactive molecules. Drawing upon the successful application of rigidification strategies in CBSI optimisation, we envisaged that integrating the imidazo[1,2-a]pyridine scaffold into the **CA-4** pharmacophore could yield novel, metabolically stable tubulin inhibitors devoid of configurational lability.

In the contemporary landscape of drug discovery, artificial intelligence (AI) and computational modelling have emerged as transformative forces that significantly accelerate the development of novel therapeutics^30–32^. AI-driven approaches, including machine learning and deep learning algorithms, have revolutionised hit identification, lead optimisation, and prediction of absorption, distribution, metabolism, and excretion (ADME) properties^33^^,^^34^. Advanced molecular docking simulations, coupled with molecular dynamics, enable high-resolution prediction of ligand-receptor interactions, guiding rational structural modifications with unprecedented precision^35^^,^^36^. Structure-based virtual screening has become an indispensable tool for efficiently navigating chemical space to identify promising candidates prior to synthesis^37–39^. Furthermore, the integration of computational predictions with experimental validation – as exemplified by our docking studies of compound **8o** within the colchicine-binding pocket – represents a paradigm for efficient, rational drug design. These cutting-edge technologies not only reduce the time and cost associated with traditional drug development but also enable the exploration of novel chemical scaffolds that would otherwise remain undiscovered. In this study, we employed molecular docking to rationalise the superior activity of **8o** and to elucidate its binding mode within the colchicine-binding site, demonstrating the value of computational approaches in guiding lead optimisation.

Herein, we present the design, synthesis, and biological evaluation of a new series of 6-aryl-3–(3,4,5-trimethoxyphenyl)imidazo[1,2-a]pyridines (**8a-u**) as novel tubulin polymerisation inhibitors. By integrating the rigid imidazo[1,2-a]pyridine core with the conserved 3,4,5-trimethoxyphenyl pharmacophore, this work aims to establish a new class of structurally stabilised CBSIs that combine potent antiproliferative activity with favourable drug-like properties, thereby offering promising lead candidates for further anticancer drug development.

## Result and discussion

### Chemistry

**Scheme 1.**
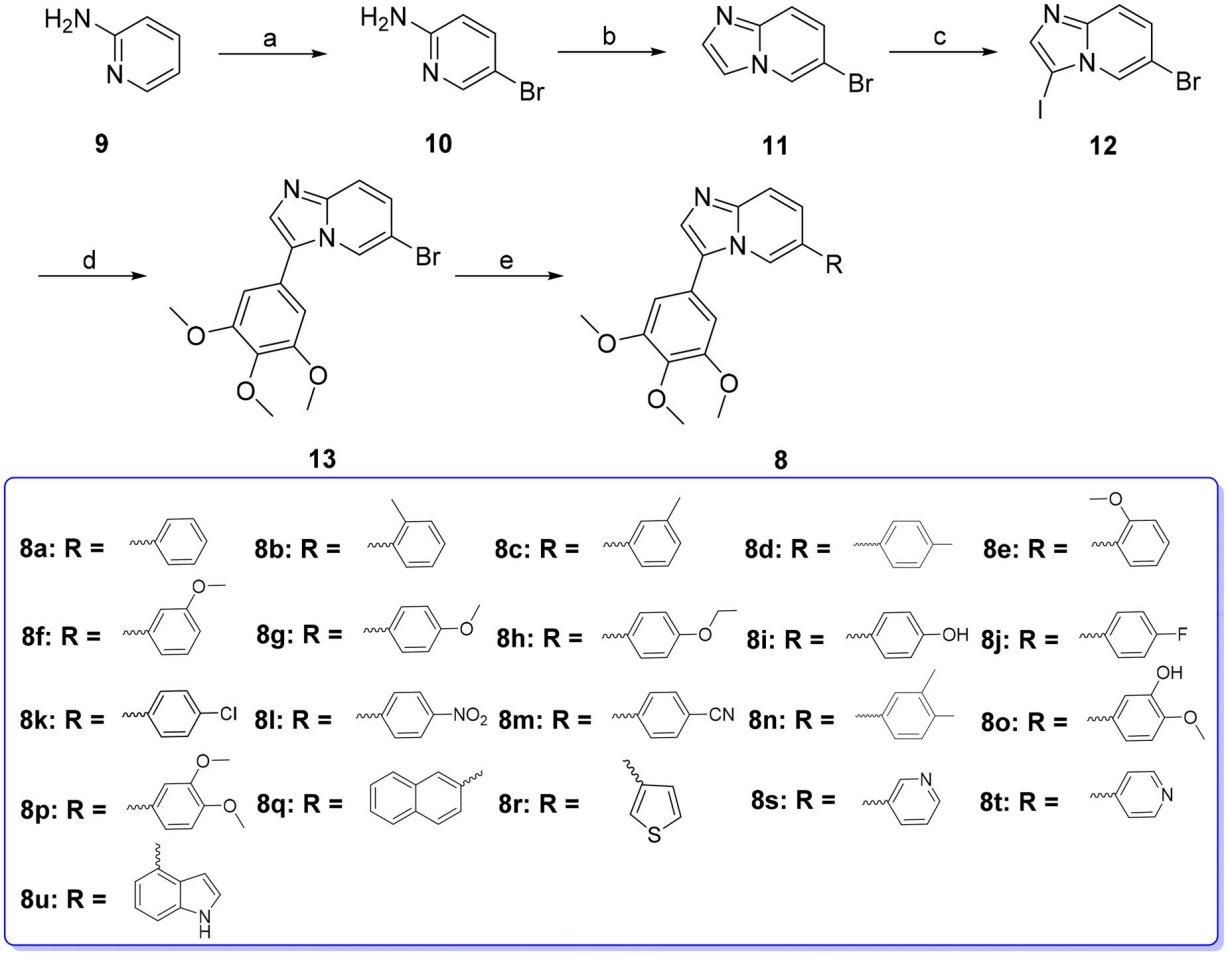
Reagents and conditions (a) NBS, MeCN, rt., 2h, 98%; (b) ClCH_2_CHO, NaHCO_3_, *i*-PrOH, 100 °C, 24 h, 80%; (c) NIS, anhydrous CH_3_CN, rt., 12h, 85%; (d) 3,4,5-trimethoxyphenylboric acid, [Pd(PPh_3_)_4_], K_2_CO_3_, 1,4-dioxane/H_2_O, N_2_ atmosphere,126 °C, microwave (M.W), 25 min, 75%; (e) Substituted phenylboronic acid, [Pd(PPh_3_)_4_], K_2_CO_3_, 1,4-dioxane/H_2_O, N_2_ atmosphere,125 °C, M.W., 26 min.

Scheme 1 illustrates the synthetic pathway for the preparation of the target 6-aryl-3–(3,4,5-trimethoxyphenyl)imidazo[1,2-a]pyridine derivatives (**8a-u**). The synthesis started with commercially available pyridin-2-amine (**9**), which underwent bromination with *N*-bromosuccinimide (NBS) in anhydrous acetonitrile at room temperature for 2 h, affording 5-bromopyridin-2-amine (**10**) in a high yield of 98%^40^.

Subsequently, compound **10** was condensed with chloroacetaldehyde in the presence of sodium bicarbonate and isopropanol. The reaction was heated to 100 °C in a sealed pressure tube for 24 h, leading to the formation of 6-bromoimidazo[1,2-a]pyridine (**11**) with an 80% yield via a cyclisation reaction^41^.

To introduce an iodine substituent at the 3-position of the imidazo[1,2-a]pyridine scaffold, compound **11** was treated with *N*-iodosuccinimide (NIS) in anhydrous acetonitrile at 0 °C, followed by stirring at room temperature for 12 h under an inert atmosphere. This iodination reaction produced 6-bromo-3-iodoimidazo[1,2-a]pyridine (**12**) as an off-white solid with an 85% yield^28^^,^^29^.

The key step for incorporating the 3,4,5-trimethoxyphenyl pharmacophore was a Suzuki cross-coupling reaction. Compound **12** was reacted with 3,4,5-trimethoxyphenylboronic acid in a mixed solvent of 1,4-dioxane and water. The reaction was catalysed by tetrakis(triphenylphosphine)palladium(0) [Pd(PPh_3_)_4_] with potassium carbonate as the base, and was carried out under microwave irradiation at 126 °C for 25 min under an argon atmosphere. This transformation afforded 6-bromo-3–(3,4,5-trimethoxyphenyl)imidazo[1,2-a]pyridine (**13**) with a 75% yield^42^.

Finally, the diversification of the 6-position of the imidazo[1,2-a]pyridine core was realised through another Suzuki cross-coupling reaction. Intermediate **13** was reacted with a variety of commercially available or synthetically accessible arylboronic acids under microwave irradiation at 125 °C for 26 min. The reaction conditions were the same as the previous cross-coupling step, using [Pd(PPh_3_)_4_] as the catalyst and potassium carbonate as the base^43^. This versatile synthetic strategy enabled the efficient preparation of the target compounds 6-aryl-3–(3,4,5-trimethoxyphenyl)imidazo[1,2-a]pyridines **(8a-u)** in moderate to excellent yields, laying the foundation for subsequent structure-activity relationship (SAR) studies.

### Biological evaluation

#### In vitro anti-proliferative activity

The antiproliferative potential of the synthesised 6-aryl-3–(3,4,5-trimethoxyphenyl)imidazo[1,2-a]pyridine derivatives (**8a-u**) was evaluated against three human cancer cell lines – HeLa (cervical carcinoma), HCT116 (colorectal carcinoma), and MCF-7 (breast carcinoma) – using the MTT assay following 72 h of continuous treatment. **CA-4**, a well-characterized colchicine-binding site inhibitor (CBSI), was employed as the positive control. The half-maximal inhibitory concentration (IC_50_) values are summarised in Table 1.

**Table 1.** *In vitro* anticancer activity (IC_50_ in μM^a^) of compounds (**8a-u**).

Compounds	(IC_50_ ± SD, μM)		
	HeLa	HCT116	MCF-7
**8a**	2.8 ± 0.53	2.2 ± 1.4	5.2 ± 4.5
**8b**	4.8 ± 1.3	4.2 ± 3.5	11 ± 13
**8c**	1.9 ± 0.85	1.7 ± 0.47	9.4 ± 2.4
**8d**	0.13 ± 0.10	0.80 ± 0.38	0.38 ± 0.14
**8e**	17 ± 3.9	20 ± 18	19 ± 4.7
**8f**	6.8 ± 1.9	4.1 ± 1.1	6.9 ± 0.14
**8g**	0.098 ± 0.040	2.0 ± 1.0	0.47 ± 0.12
**8h**	5.9 ± 2.1	13 ± 2.6	7.0 ± 1.5
**8i**	39 ± 21	>100	45 ± 30
**8j**	8.2 ± 1.1	23 ± 22	14 ± 4.6
**8k**	3.1 ± 0.89	9.5 ± 1.3	3.5 ± 2.3
**8l**	59 ± 26	>100	20 ± 15
**8m**	20 ± 8.3	39 ± 2.2	21 ± 6.6
**8n**	0.45 ± 0.25	0.91 ± 1.0	0.50 ± 0.37
**8o**	**0.050 ± 0.021**	**0.069 ± 0.042**	**0.078 ± 0.051**
**8p**	0.12 ± 0.08	0.31 ± 0.28	0.41 ± 0.06
**8q**	3.3 ± 1.5	16 ± 15	2.7 ± 2.3
**8r**	0.19 ± 0.15	9.2 ± 0.38	0.14 ± 0.17
**8s**	9.2 ± 6.5	32 ± 2.9	10 ± 9.2
**8t**	10 ± 1.8	32 ± 7.2	9.3 ± 6.1
**8u**	0.096 ± 0.023	0.45 ± 0.12	0.46 ± 0.42
**CA-4** ^b^	0.045 ± 0.022	0.055 ± 0.033	0.063 ± 0.026

^a^IC_50_: the half maximal inhibitory concentration. All IC_50_ values are presented as mean ± standard deviation (SD) from three independent experiments (*n* = 3), each performed in triplicate.

^b^Used as positive controls.

Most of the synthesised compounds exhibited significant antiproliferative activity, thereby validating the rational design strategy of rigidifying the **CA-4**-like pharmacophore within the imidazo[1,2-a]pyridine scaffold. Among all derivatives, compound **8o**, bearing a 2-methoxy-5-hydroxyphenyl moiety at the 6-position, demonstrated the most potent inhibitory effects across all tested cell lines, with IC_50_ values of **0.050 ± 0.021 μM** (HeLa), 0.069 ± 0.042 μM (HCT116), and 0.078 ± 0.051 μM (MCF-7). These values are comparable to those of **CA-4** (IC_50_ = 0.045 ± 0.022 μM for HeLa, 0.055 ± 0.033 μM for HCT116, and 0.063 ± 0.026 μM for MCF-7), highlighting **8o** as a highly promising analogue. In addition, several other derivatives, including **8d** (*p*-tolyl), **8 g** (*p*-methoxyphenyl), **8n** (3,4-dimethylphenyl), **8p** (3,4-dimethoxyphenyl), **8r** (thiophen-3-yl), and **8 u** (1*H*-indol-4-yl), also exhibited potent antiproliferative activity with IC_50_ values below 1 μM in at least one cell line.

Preliminary structure-activity relationship (SAR) analysis revealed that the position and electronic nature of substituents on the 6-aryl ring significantly influenced potency. Para-substituted electron-donating groups (e.g. methyl in **8d**, methoxy in **8 g**) generally enhanced activity, whereas ortho- or meta-substitution, as well as electron-withdrawing groups (e.g. fluoro, chloro, nitro, cyano), led to reduced potency. Moreover, heteroaromatic substitutions such as thiophene (**8r**) and indole (**8 u**) were well tolerated, while naphthyl (**8q**) and pyridinyl (**8s**, **8t**) analogues showed diminished activity, suggesting that both the geometry and electronic characteristics of the 6-aryl substituent are critical for optimal interaction with the colchicine-binding site. Statistical significance was evaluated using one-way analysis of variance followed by Tukey’s *post hoc* test. A *p* values < 0.05 was considered statistically significant. The analysis revealed that compound **8o** exhibited comparable potency to the positive control **CA-4** across all three cell lines (*p* > 0.05), while demonstrating significantly higher antiproliferative activity than less active derivatives such as **8 g**, **8p**, and **8 u** (*p* < 0.01).

To further evaluate the selectivity of **8o**, its cytotoxicity was assessed against human umbilical vein endothelial cells (HUVECs), a normal cell model. As shown in Table 2, **8o** exhibited significantly lower toxicity towards HUVECs (IC_50_ = 3.1 μM) compared to **CA-4** (IC_50_ = 1.0 μM), resulting in an approximately 3-fold higher safety margin. This selective cytotoxicity, combined with its potent antiproliferative activity against cancer cells, underscores the therapeutic potential of **8o** as a lead compound with an improved therapeutic index.

**Table 2.** *In vitro* anticancer activity and cytotoxicity test of **8o** and **CA-4**.

Compounds	(IC_50_, μM)^a^
	HUVECs
**8o**	3.1
**CA-4**	1.0

^a^IC_50_: the half maximal inhibitory concentration.

In summary, the imidazo[1,2-a]pyridine scaffold effectively stabilises the bioactive conformation of the **CA-4**-like pharmacophore, and appropriate substitution at the 6-position can further enhance antiproliferative potency and selectivity. The outstanding performance of **8o** validates the scaffold-hopping strategy and supports its advancement for further mechanistic and preclinical evaluation.

#### Effect on tubulin polymerisation

To directly evaluate the ability of compound **8o** to modulate microtubule dynamics, an *in vitro* tubulin polymerisation assay was performed according to established protocols^44^. Paclitaxel (PTX), a known microtubule-stabilising agent, and **CA-4**, a potent microtubule-destabilizer, were used as negative and positive controls, respectively.

**Figure 3.**
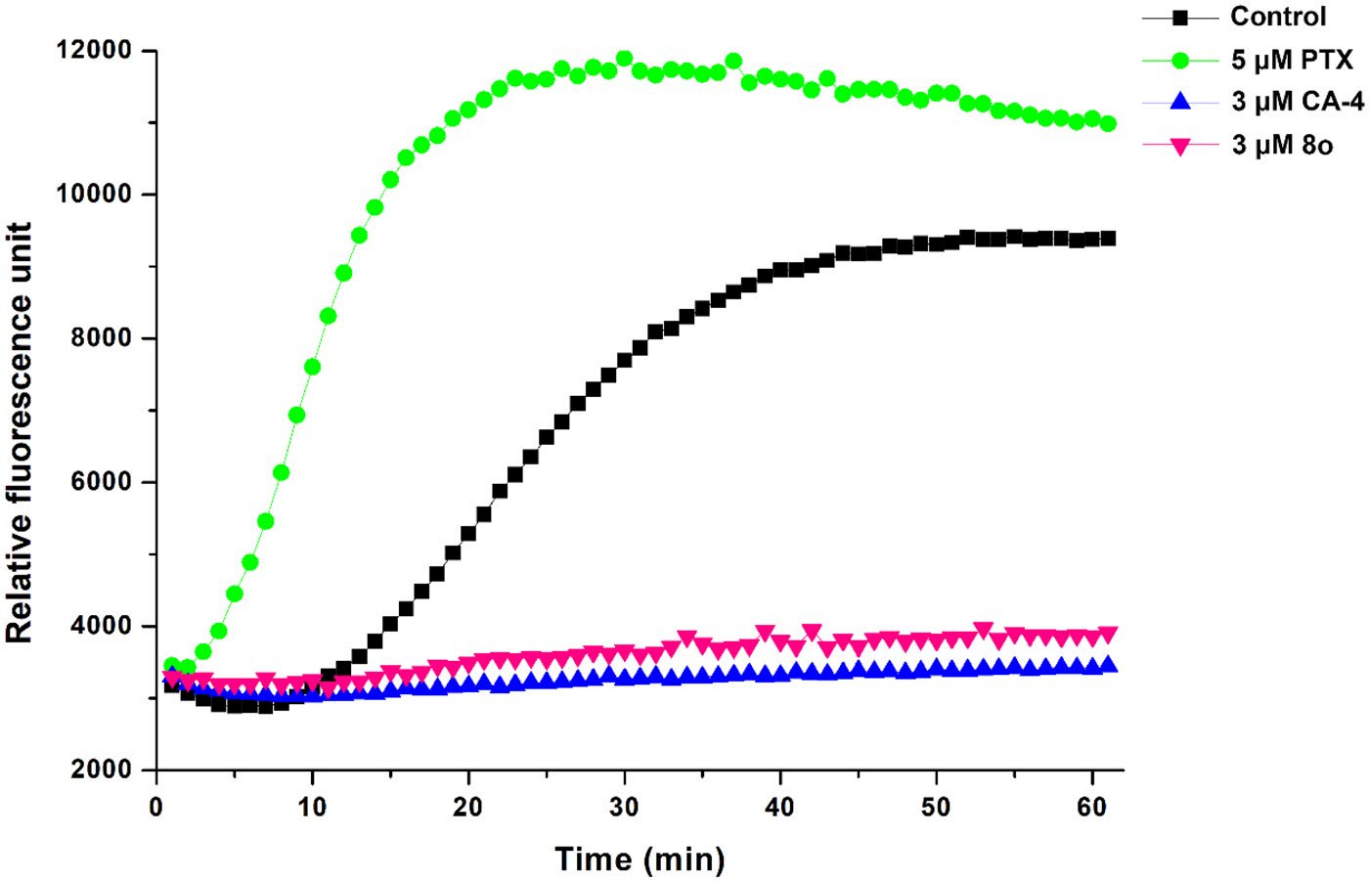
Effects of compound **8o** and control compounds on tubulin polymerisation.

As illustrated in Figure 3, PTX markedly accelerated tubulin polymerisation relative to the vehicle control, consistent with its stabilising mechanism. In contrast, both **8o** and **CA-4** effectively suppressed the polymerisation kinetics, indicating a destabilising effect on microtubule assembly. Notably, at an equimolar concentration of 3 μM, **8o** exhibited an inhibitory potency comparable to that of **CA-4**, confirming its direct and potent interference with tubulin polymerisation.

Collectively, these results demonstrate that **8o** functions as a potent microtubule-destabilising agent, with efficacy comparable to the benchmark CBSI **CA-4**. This finding reinforces the potential of the imidazo[1,2-a]pyridine scaffold as a promising chemotype for the development of novel and potent tubulin polymerisation inhibitors, warranting further structural optimisation and mechanistic investigation.

#### Analysis of immunofluorescence staining

**Figure 4.**
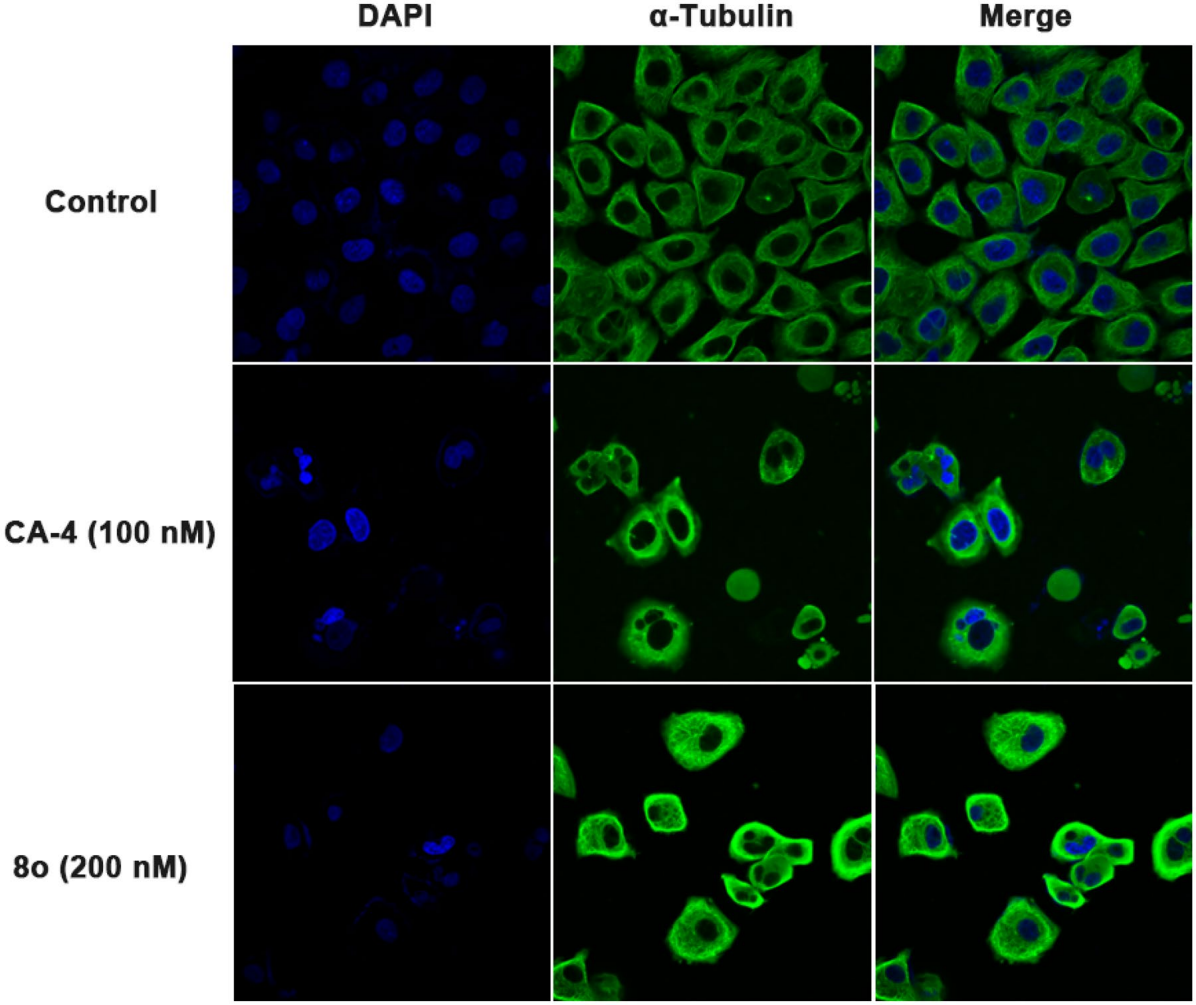
Immunofluorescence analysis showing the effects of compound **8o** and **CA-4** on the cellular microtubule network and microtubule reorganisation.

To further characterise the cellular impact of compound **8o** on microtubule organisation, immunofluorescence microscopy was performed in HeLa cells following established protocols^43^. As shown in Figure 4, untreated control cells displayed a well-organised, extensive filamentous microtubule network with uniformly stained nuclei, indicative of an intact cytoskeletal architecture.

In contrast, treatment with **8o** at 200 nM for 24 h led to profound disruption of the microtubule network. The filamentous structures became fragmented and collapsed, forming dense, perinuclear aggregates that circumscribed the nucleus – a hallmark of microtubule depolymerisation. Additionally, pronounced nuclear pyknosis, reflecting chromatin condensation, and the appearance of multinucleated cells, indicative of mitotic defects and failed cytokinesis, were readily observed. Notably, **CA-4** at 100 nM induced a nearly identical phenotype, with comparable microtubule disassembly, perinuclear aggregation, nuclear pyknosis, and multinucleation, demonstrating that the effects of **8o** at 200 nM and **CA-4** at 100 nM are essentially equivalent.

These results provide direct cellular evidence that **8o** potently destabilises microtubules and disrupts normal mitotic progression. The phenotypic concordance with **CA-4** strongly supports a shared mechanism of action involving binding to the colchicine site on β-tubulin, and further validates **8o** as a highly effective microtubule-destabilising agent capable of inducing mitotic catastrophe and multinucleation in cancer cells.

#### Cell cycle analysis

Antimitotic agents represent a cornerstone of anticancer therapy, primarily functioning through inhibition of tubulin polymerisation and disruption of microtubule dynamics, which culminates in cell cycle arrest at the G_2_/M phase and suppression of tumour proliferation^42^. To assess whether compound **8o** exhibits such antimitotic properties, its effect on cell cycle distribution was evaluated in HeLa cells using flow cytometry after 24 h of treatment.

**Figure 5.**
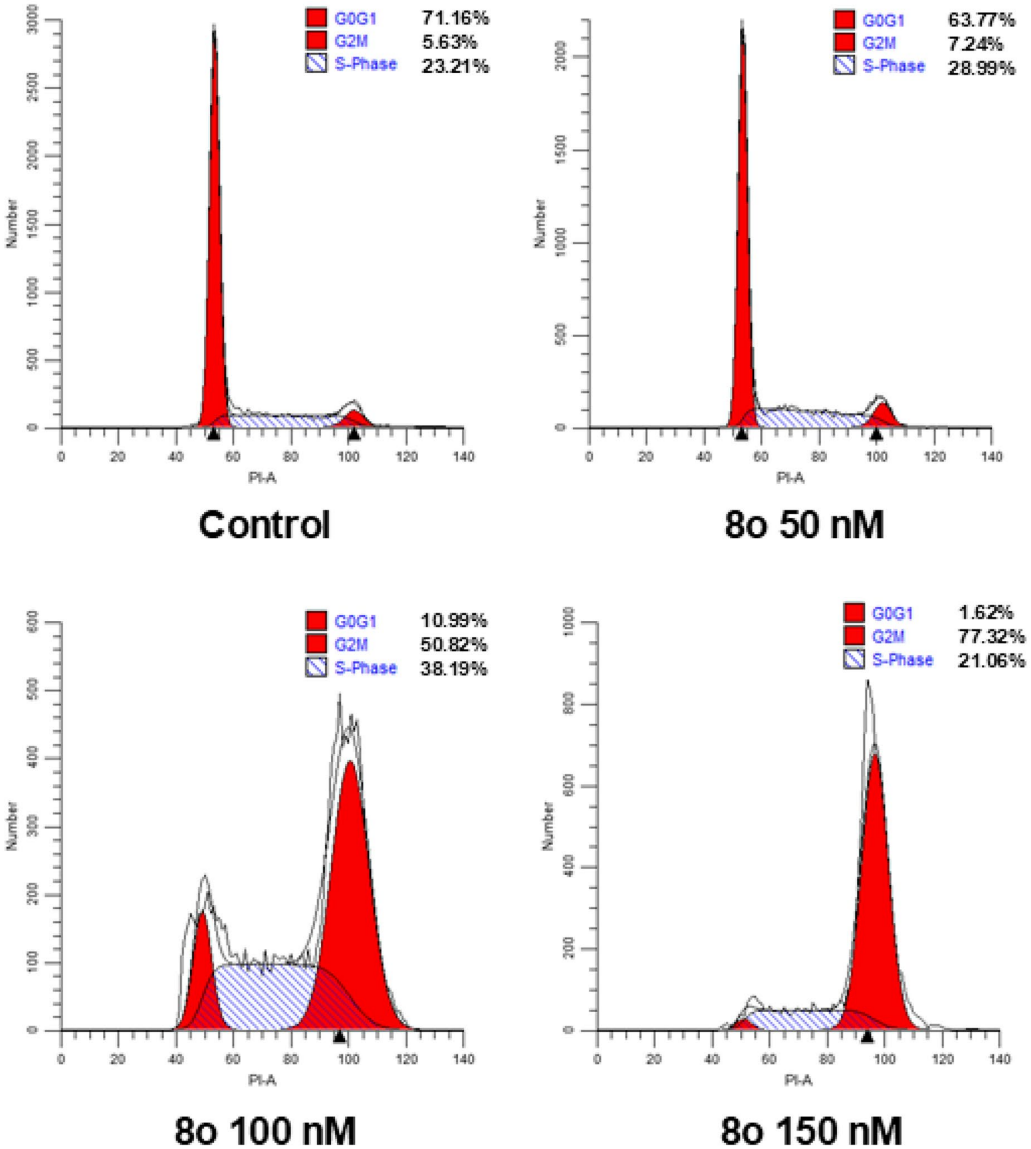
Effects of compound **8o** on the cell cycle distribution in HeLa cells.

As shown in Figure 5, exposure to **8o** across a concentration range of 50-150 nM resulted in a dose-dependent and substantial accumulation of cells in the G_2_/M phase. The proportion of G_2_/M-arrested cells increased from 5.63% in the control group to 77.32% at the highest concentration tested. This pronounced G_2_/M arrest indicates that **8o** effectively disrupts mitotic progression, consistent with the behaviour of established microtubule-targeting agents.

The robust cell cycle blockade induced by **8o** aligns well with its previously demonstrated inhibition of tubulin polymerisation and microtubule destabilisation, supporting a coherent mechanism wherein cytoskeletal disruption translates into mitotic arrest. These results further substantiate the antiproliferative potential of **8o** and reinforce its profile as a promising candidate within the class of colchicine-site tubulin inhibitors. Future studies aimed at delineating the downstream signalling events triggered by **8o** may provide valuable insights for the development of novel mitotic inhibitors with improved therapeutic efficacy.

#### Induction of cell apoptosis

Microtubule-targeting agents that bind the colchicine site are recognised for their capacity to initiate programmed cell death in cancer cells^43^. To assess the apoptotic activity of compound **8o**, HeLa cells were exposed to a concentration gradient of **8o** for 48 h, followed by evaluation of apoptosis using Annexin V-FITC/propidium iodide dual-staining and flow cytometric analysis.

**Figure 6.**
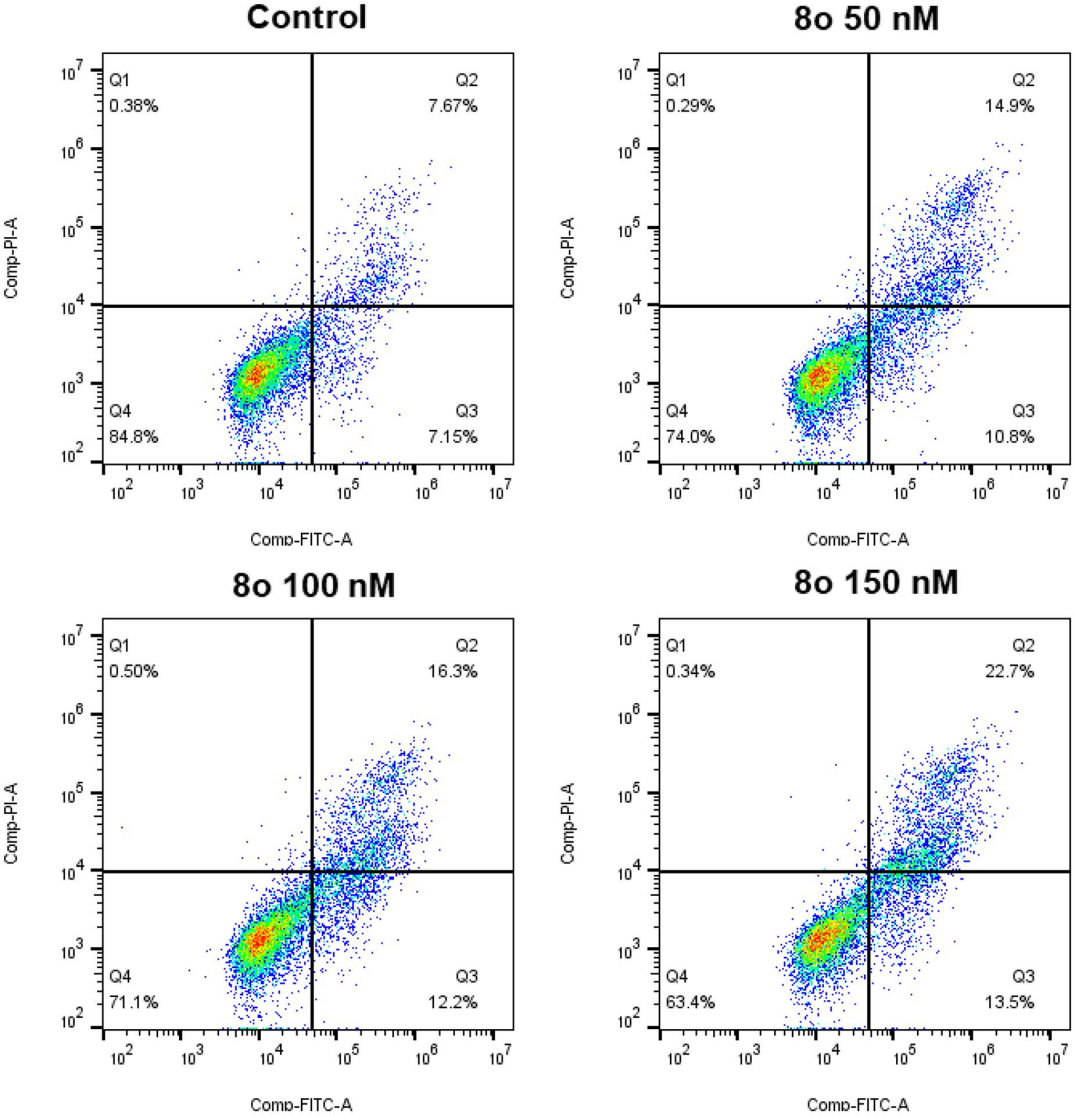
Analysis of apoptosis induction by compound **8o** in HeLa cells.

As summarised in Figure 6, **8o** triggered a clear concentration-dependent induction of apoptosis. Treatment with 50 nM **8o** led to a modest apoptotic rate of 25.7%, whereas concentrations of 100 nM and 150 nM increased apoptosis to 28.5% and 36.2%, respectively. These data demonstrate that **8o** effectively promotes apoptosis in a dose-responsive manner in hepatocellular carcinoma cells.

The ability of **8o** to elicit apoptosis aligns with the recognised cytotoxic mechanism of CBSIs and complements its previously observed anti-mitotic and microtubule-destabilising effects. The progressive induction of cell death supports the potential of **8o** as a promising lead compound in this series. Further studies aimed at elucidating the specific apoptotic pathways – such as mitochondrial-mediated or death-receptor-driven signalling – activated by **8o**, as well as its efficacy in *in vivo* models, are warranted to advance its preclinical development for cervical cancer treatment.

### Molecular modelling study

**Figure 7.**
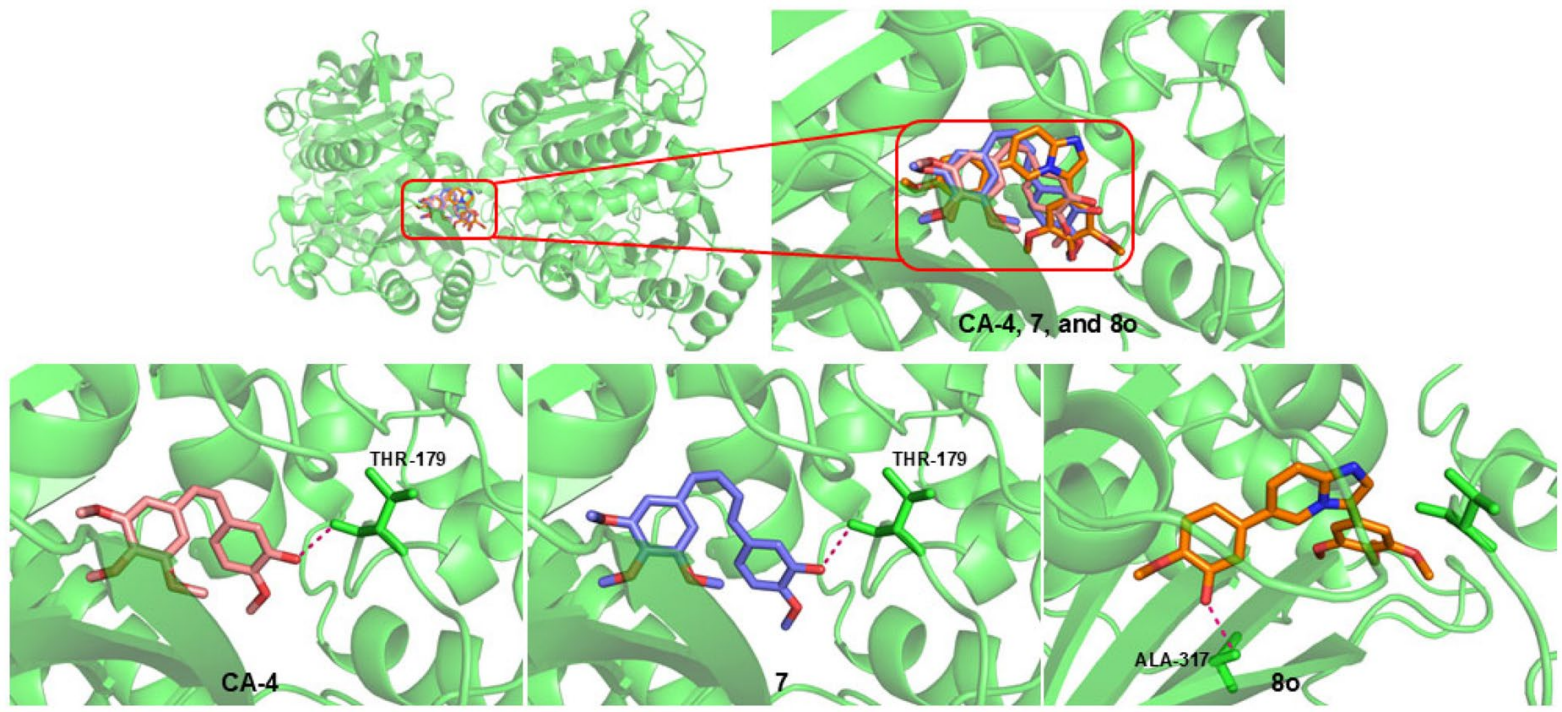
Proposed binding modes for **8o** in comparison with **CA-4** and **7** at the colchicine-site. Carbon atoms are shown in pale red for **CA-4**, in purple for **7**, and in ginger for **8o**. The residues from the *α*, *β*-tubulin chain are shown in green.

To rationalise the potent biological activity of compound **8o** at the molecular level, a computational docking study (Figure 7) was conducted using the high-resolution crystal structure of tubulin complexed with a colchicine-site ligand (PDB: 5LYJ)^43^. The predicted binding conformation of **8o** was analysed and compared with those of the reference CBSIs CA‑4 and compound **7** to identify critical intermolecular interactions. The docking simulation revealed that **8o** occupies the colchicine-binding pocket in a well-defined orientation, with its 3,4,5‑trimethoxyphenyl moiety engaging in characteristic hydrophobic contacts with residues such as *β*‑Lys352, *β*‑Asn349, and *β*‑Asn258. More notably, a specific and energetically favourable hydrogen bond was formed between the hydroxyl group of **8o** and the backbone carbonyl of *β*‑Ala317 – an interaction not observed in the binding poses of CA‑4 or **7**. This additional polar contact is likely to enhance binding affinity and may contribute to the improved inhibitory potency of **8o** relative to the reference compounds. Furthermore, the rigid imidazo[1,2‑a]pyridine core of **8o** appears to be optimally positioned to maintain the spatial arrangement of the two aryl rings required for effective binding, while preventing the configurational isomerisation that limits the stability of flexible analogues. These computational findings provide a structural explanation for the enhanced antiproliferative activity of **8o**.

Based on the combined SAR and docking analyses, several rational optimisation strategies can be proposed for further development of this imidazo[1,2-a]pyridine series. First, the 6-position of the core scaffold tolerates a range of substituents, with the 2-methoxy-5-hydroxyphenyl group in **8o** conferring the highest potency. The hydrogen bond between the 5-hydroxy group and the backbone carbonyl of *β*-Ala317, identified in docking studies, suggests that introducing additional hydrogen-bond donors or acceptors at this region could further enhance binding affinity. Second, the conserved 3,4,5-trimethoxyphenyl pharmacophore remains essential for hydrophobic anchoring within the colchicine pocket; however, modification of the methoxy groups to other bioisosteres may improve metabolic stability without compromising activity. Third, the planar imidazo[1,2-a]pyridine core successfully locks the bioactive conformation, but further rigidification *via* heterocyclic ring fusion could potentially enhance selectivity for the colchicine site over other tubulin-binding pockets. Lastly, the favourable drug-like properties of **8o**, as indicated by Lipinski’s rule of five, support further lead optimisation efforts focusing on improving aqueous solubility and pharmacokinetic profiles while maintaining potent antiproliferative activity. These strategies collectively provide a roadmap for the rational design of next-generation tubulin inhibitors with improved therapeutic potential.

### Physicochemical properties

To assess the drug-likeness and potential oral bioavailability of the synthesised compounds, key physicochemical parameters were predicted in accordance with Lipinski’s rule of five. For the representative compound **8o** and the reference CBSI CA‑4, molecular descriptors – including molecular weight (MW), calculated lipophilicity (CLog P), number of hydrogen bond donors (HBD) and acceptors (HBA), and topological polar surface area (TPSA) – were determined using SwissADME (http://www.swissadme.ch) and ChemBioDraw Ultra 14.0.

As presented in Table 3, all evaluated compounds comply fully with Lipinski’s criteria, suggesting acceptable drug-like properties. Notably, **8o** displays a favourable balance of molecular size, lipophilicity, and polar surface area, aligning with the profile of established orally bioavailable drugs.

**Table 3.** Predicted physicochemical properties of **CA-4** and **8o**.^a^

Compounds	CLogP	TPSA	Natoms	MW	HBA	HBD
Standard	< 5	< 140		< 500	< 10	< 5
**CA-4**	3.32	57.15	43	316.13	4	1
**8o**	3.98	72.75	52	406.44	6	1

^a^CLogP: Calculated LogP; TPSA: topological polar surface area; Natoms: No. of atoms; MW: molecular weight; HBA: hydrogen-bond acceptor atoms. HBD: hydrogen-bond donor atoms.

It is important to note, however, that these basic physicochemical parameters represent only an initial assessment of drug-likeness and are not sufficient to definitively predict oral bioavailability or pharmacokinetic behaviour. More comprehensive evaluations – including advanced *in silico* models (e.g. predictions of gastrointestinal absorption, P-glycoprotein substrate status, and physiologically based pharmacokinetic modelling) as well as experimental pharmacokinetic studies – are necessary to fully characterise the ADME properties of **8o**. Such investigations are planned as part of our ongoing research and will be reported in due course. Collectively, the current preliminary data provide initial support for the continued development of **8o** as a promising lead candidate, while recognising that further pharmacokinetic characterisation is warranted.

## Conclusion

In this study, a focused series of novel 6-aryl-3–(3,4,5-trimethoxyphenyl)imidazo[1,2-a]pyridine derivatives was designed and synthesised based on a scaffold-hopping strategy targeting the colchicine-binding site of tubulin. Through systematic *in vitro* evaluation, the majority of these compounds demonstrated potent antiproliferative activities against a panel of human cancer cell lines (HeLa, HCT116, and MCF-7), with compound **8o** exhibiting the most pronounced cytotoxic effects, displaying IC_50_ values in the nanomolar range (0.050-0.078 µM).

Mechanistic studies revealed that **8o** functions as a potent inhibitor of tubulin polymerisation in a biochemical assay. This primary mechanism was further corroborated by cellular observations, in which **8o** induced marked disruption of microtubule networks, arrested the cell cycle at the G_2_/M phase, and subsequently promoted apoptotic cell death. Molecular docking analysis supports a high-affinity interaction of **8o** within the colchicine-binding pocket, with predicted binding free energies that compare favourably with known CBSIs.

Notably, compound **8o** possesses favourable drug-like characteristics, complying with Lipinski’s rule of five, which provides initial support for its potential as a drug-like lead candidate. However, it should be emphasised that these basic physicochemical parameters represent only a preliminary assessment; comprehensive experimental pharmacokinetic studies and advanced *in silico* modelling are necessary to fully evaluate the oral bioavailability and ADME properties of **8o**. Such investigations are planned for future work. Collectively, these results establish **8o** as a structurally stabilised, potent, and metabolically robust colchicine-site tubulin inhibitor with compelling antitumor activity. These findings not only validate the effectiveness of the imidazo[1,2-a]pyridine-based rigidification strategy in overcoming the configurational instability inherent in earlier CBSIs, but also highlight **8o** as a promising lead candidate warranting further preclinical development towards potential anticancer therapeutics.

Looking forward, several specific research directions warrant further exploration to advance this class of tubulin inhibitors towards clinical candidacy. First, comprehensive *in vivo* pharmacokinetic and pharmacodynamic studies of compound **8o** are essential to evaluate its bioavailability, tissue distribution, metabolic stability, and antitumor efficacy in appropriate xenograft models. Second, guided by the structure-activity relationships and molecular docking insights presented herein, further structural optimisation will focus on modulating the 6-aryl substituent to enhance hydrogen-bonding interactions within the colchicine-binding pocket, as exemplified by the hydroxyl group in **8o**, while exploring bioisosteric replacements for the trimethoxyphenyl moiety to improve metabolic stability. Third, mechanistic investigations aimed at delineating the precise apoptotic pathways – including mitochondrial dysfunction, caspase activation, and Bcl-2 family modulation – will provide deeper insights into the molecular basis of **8o**-induced cell death. Fourth, the potential for synergistic combination of **8o** with established chemotherapeutics, such as taxanes or platinum-based agents, will be assessed to explore opportunities for overcoming drug resistance and enhancing therapeutic efficacy. Collectively, these efforts will establish a robust foundation for the continued development of imidazo[1,2-a]pyridine-based tubulin inhibitors as promising anticancer agents.

## Experimental

### Chemistry

#### Materials and methods

All commercially available chemicals and solvents were used without further purification unless otherwise specified. Routine monitoring of reactions was performed using silica gel 60 GF254 thin-layer chromatography plates (Energy Chemical, Anhui, China), with UV light (254 nm) for visualisation. Column chromatography purifications were carried out on silica gel (100-200 mesh, Aladdin, Shanghai, China).^1^H and 1³C NMR spectra were recorded at 25 °C on a Bruker AVANCE III HD 500 spectrometer operating at 500 MHz for ^1^H and 126 MHz for 1³C. Samples were dissolved in deuterated chloroform (CDCl_3_, Aladdin, Shanghai, China) or dimethyl sulfoxide-d_6_ (DMSO-d_6_, Aladdin, Shanghai, China) with tetramethylsilane (TMS) as internal reference. Chemical shifts (*δ*) are reported in parts per million (ppm), and coupling constants (J) are given in Hertz (Hz). High-resolution mass spectrometry (HR-MS) was performed on an Agilent 6530 Accurate-Mass Q-TOF LC/MS system equipped with an electrospray ionisation (ESI) source operating in positive ion mode. Selected microwave-assisted reactions were conducted in a CEM Discover SP single-mode microwave reactor. All human cancer cell lines used in this study were obtained from the Cell Bank of the Chinese Academy of Sciences (Shanghai, China) and maintained according to the supplier’s recommendations.

#### General synthetic procedure for 5-bromopyridin-2-amine (10)

Pyridin-2-amine (**9**, 2.6 g, 27.6 mmol) was dissolved in anhydrous acetonitrile (40 mL). To this solution, *N*-bromosuccinimide (5.2 g, 29.2 mmol) was added portionwise at room temperature. The resulting mixture was stirred under an inert atmosphere for 2 h while monitoring by thin-layer chromatography. Upon completion, the solvent was evaporated under reduced pressure. The crude product was purified by flash column chromatography (silica gel; eluent: petroleum ether/ethyl acetate, 8:2, *v*/*v*) to afford **10** as a white crystalline solid (4.7 g, 98%).

#### General synthetic procedure for 6-bromoimidazo[1,2-a]pyridine (11)

A mixture of 5-bromopyridin-2-amine (**10**, 2.8 g, 16.1 mmol), chloroacetaldehyde (1.5 mL, 19.3 mmol), NaHCO_3_ (2.7 g, 32.2 mmol), and isopropanol (10 mL) was placed in a sealed pressure tube. The reaction mixture was heated to 100 °C under stirring and maintained for 24 h. After cooling to room temperature, the solvent was removed under reduced pressure. The crude residue was taken up in dichloromethane (30 mL) and washed sequentially with water (20 mL × 2) and brine (20 mL). The aqueous layers were back‑extracted with dichloromethane (20 mL × 2). The combined organic extracts were dried over anhydrous MgSO_4_, filtered, and concentrated *in vacuo*. The resulting solid was recrystallized from hexane to afford **11** as a pale‑yellow crystalline solid (2.5 g, 80%).

#### General synthetic procedure for 6-bromo-3-iodoimidazo[1,2-a]pyridine (12)

To a solution of **11** (1.1 g, 5.4 mmol) in anhydrous acetonitrile (15 mL) was added *N*-iodosuccinimide (1.2 g, 5.4 mmol) portionwise at 0 °C. The reaction mixture was then allowed to warm to room temperature and stirred for 12 h under an inert atmosphere, with the progress monitored by thin-layer chromatography. Upon completion, the mixture was concentrated under reduced pressure. The resulting crude solid was triturated with cold acetonitrile (10 mL), collected by filtration, and washed with additional cold acetonitrile (5 mL × 2) to give **12** as an off-white solid (1.5 g, 85%).

#### General synthetic procedure for 6-bromo-3–(3,4,5-trimethoxyphenyl)imidazo[1,2-a]pyridine (13)

A mixture of **12** (0.96 g, 3.0 mmol), 3,4,5-trimethoxyphenylboronic acid (0.58 g, 2.7 mmol), K_2_CO_3_ (0.41 g, 3.0 mmol), and [Pd(PPh_3_)_4_] (35 mg, 0.03 mmol) in 1,4-dioxane (10 mL) and water (3 mL) was degassed with argon for 10 min. The reaction vessel was then sealed and subjected to microwave irradiation at 126 °C for 25 min under controlled power. After cooling to room temperature, the mixture was diluted with water (20 mL) and extracted with ethyl acetate (3 × 12 mL). The combined organic extracts were washed with brine (15 mL), dried over anhydrous Na_2_SO_4_, filtered, and concentrated under reduced pressure. The crude residue was purified by flash column chromatography (silica gel, eluent: petroleum ether/ethyl acetate, 6:4, *v*/*v*) to yield **13** as a light-yellow solid (0.82 g, 75%).

##### General synthetic procedure for 6-aryl-3–(3,4,5-trimethoxyphenyl)imidazo[1,2-a]pyridines (8a-u)

A microwave vial was charged with 6-bromo-3-(3,4,5-trimethoxyphenyl)imidazo[1,2-a]pyridine (**13**, 36 mg, 0.1 mmol), the corresponding arylboronic acid (0.15 mmol), [Pd(PPh_3_)_4_] (7.0 mg, 0.006 mmol), and K_2_CO_3_ (69 mg, 0.5 mmol). The solids were dissolved in a degassed mixture of 1,4-dioxane (5 mL) and water (1.5 mL). The vial was sealed and subjected to microwave irradiation at 125 °C for 26 min. After cooling to ambient temperature, the reaction mixture was diluted with water (10 mL) and extracted with ethyl acetate (3 × 12 mL). The combined organic layers were washed with brine (10 mL), dried over anhydrous Na_2_SO_4_, filtered, and concentrated under reduced pressure. The crude product was purified by flash column chromatography (silica gel, 100–200 mesh) using an appropriate eluent system to afford the target compounds **8a-u** in analytically pure form.

##### 6-phenyl-3–(3,4,5-trimethoxyphenyl)imidazo[1,2-a]pyridine (8a)

Yield: 77%; M.p. 116.0–116.9 °C; ^1^H NMR (500 MHz, CDCl_3_) *δ* 8.48 (s, 1H, imidazole-H), 7.65 (d, *J* = 1.4 Hz, 3H, ArH), 7.57 (d, *J* = 1.3 Hz, 1H, ArH), 7.46-7.43 (m, 3H, ArH), 7.40 (t, *J* = 7.2 Hz, 1H, ArH), 6.78 (s, 2H, ArH-2′,6′), 3.94 (s, 3H, OCH_3_-4′), 3.91 (s, 6H, OCH_3_-3′,5′); ^13^C NMR (126 MHz, CDCl_3_) *δ* 154.02 (2 C, ArC-3′,5′), 150.83, 144.63, 137.97, 133.04, 129.67, 129.24 (2 C, ArC-3,5), 128.08, 126.97 (2 C, ArC-2,6), 125.93, 125.50, 120.80, 115.32, 108.46, 105.82 (2 C, ArC-2′,6′), 61.05, 56.42 (2 C, OCH_3_-3′,5′). HRMS calcd for C_21_H_20_N_3_O_3_ [M + H]^+^ 361.1552, found 361.1548.

##### 6-(o-tolyl)-3–(3,4,5-trimethoxyphenyl)imidazo[1,2-a]pyridine (8b)

Yield: 85%; M.p. 103.4–104.8 °C; ^1^H NMR (500 MHz, CDCl_3_) *δ* 8.23 (s, 1H, imidazole-H), 7.66 (d, *J* = 7.0 Hz, 1H, ArH), 7.54 (dd, *J* = 7.4, 1.4 Hz, 1H, ArH), 7.49-7.46 (m, 2H, ArH), 7.30 (d, *J* = 1.4 Hz, 1H, ArH), 7.24 (d, *J* = 1.6 Hz, 1H, ArH), 7.22 (dd, *J* = 9.2, 1.6 Hz, 1H, ArH), 6.76 (s, 2H, ArH-2′,6′), 3.90 (s, 3H, OCH_3_-4′), 3.89 (s, 6H, OCH_3_-3′,5′), 2.33 (s, 3H, ArCH_3_); ^13^C NMR (126 MHz, CDCl_3_) *δ* 153.91 (2 C, ArC-3′,5′), 145.06, 138.32, 137.54, 135.89, 133.05, 132.67, 130.68, 129.93, 128.22, 127.28, 127.03, 126.18, 124.65, 122.28, 117.36, 105.78 (2 C, ArC-2′,6′), 61.00, 56.35 (2 C, OCH_3_-3′,5′), 20.48. HRMS calcd for C_23_H_23_N_2_O_3_ [M + H]^+^ 375.1709, found 375.1708.

##### 6-(m-tolyl)-3–(3,4,5-trimethoxyphenyl)imidazo[1,2-a]pyridine (8c)

Yield: 84%; M.p. 62.8–64.7 °C; ^1^H NMR (500 MHz, CDCl_3_) *δ* 8.46 (s, 1H, imidazole-H), 7.73 (d, *J* = 9.3 Hz, 1H, ArH), 7.65 (s, 1H, ArH), 7.55 (t, *J* = 6.8 Hz, 2H, ArH), 7.45 (d, *J* = 2.8 Hz, 1H, ArH), 7.36 (d, *J* = 7.5 Hz, 1H, ArH), 7.21 (d, *J* = 6.6 Hz, 1H, ArH), 6.79 (s, 2H, ArH-2′,6′), 3.94 (s, 3H, OCH_3_-4′), 3.91 (s, 6H, OCH_3_-3′,5′), 2.42 (s, 3H, ArCH_3_); ^13^C NMR (126 MHz, CDCl_3_) *δ* 153.97 (2 C, ArC-3′,5′), 145.31, 138.90, 138.29, 137.46, 133.04, 132.79, 129.08, 128.70, 127.74, 127.30, 126.11, 125.19, 124.70, 124.02, 120.66, 118.02, 105.70 (2 C, ArC-2′,6′), 61.04, 56.38 (2 C, OCH_3_-3′,5′), 22.04. HRMS calcd for C_23_H_23_N_2_O_3_ [M + H]^+^ 375.1709, found 375.1708.

##### 6-(p-tolyl)-3–(3,4,5-trimethoxyphenyl)imidazo[1,2-a]pyridine (8d)

Yield: 73%; M.p. 120.0–121.3 °C; ^1^H NMR (500 MHz, CDCl_3_) *δ* 8.46 (s, 1H, imidazole-H), 7.72 (d, *J* = 9.3 Hz, 1H, ArH), 7.55-7.53 (m, 2H, ArH), 7.43 (d, *J* = 8.2 Hz, 2H, ArH-2″,6″), 7.27 (d, *J* = 7.8 Hz, 2H, ArH-3″,5″), 6.78 (s, 2H, ArH-2′,6′), 3.94 (s, 3H, OCH_3_-4′), 3.91 (s, 6H, OCH_3_-3′,5′), 2.40 (s, 3H, ArCH_3_); ^13^C NMR (126 MHz, CDCl_3_) *δ* 153.96 (2 C, ArC-3′,5′), 145.26, 138.26, 137.88, 134.58, 133.03, 132.75, 129.88 (2 C, ArC-3″,5″), 127.12, 126.76 (2 C, ArC-2″,6″), 125.12, 124.74, 120.38, 118.02, 105.68 (2 C, ArC-2′,6′), 61.04, 56.39 (2 C, OCH_3_-3′,5′), 21.14. HRMS calcd for C_23_H_23_N_2_O_3_ [M + H]^+^ 375.1709, found 375.1709.

##### 6-(2-methoxyphenyl)-3-(3,4,5-trimethoxyphenyl)imidazo[1,2-a]pyridine (8e)

Yield: 91%; M.p. 58.9–60.1 °C;^1^H NMR (500 MHz, CDCl_3_) *δ* 8.45 (s, 1H, imidazole-H), 7.65 (d, *J* = 1.4 Hz, 1H, ArH), 7.46–7.44 (m, 1H, ArH), 7.42 (dd, *J* = 9.3, 1.5 Hz, 1H, ArH), 7.40-7.34 (m, 1H, ArH), 7.31 (dd, *J* = 7.5, 1.6 Hz, 1H, ArH), 7.03 (dd, *J* = 15.4, 7.9 Hz, 2H, ArH), 6.78 (s, 2H, ArH-2′,6′), 3.92 (s, 3H, OCH_3_-4′), 3.90 (s, 6H, OCH_3_-3′,5′), 3.84 (s, 3H, OCH_3_-2″); ^13^C NMR (126 MHz, CDCl_3_) *δ* 156.67, 153.87 (2 C, ArC-3′,5′), 145.16, 138.21, 133.05, 132.48, 130.55, 129.48, 127.39, 126.56, 124.86, 124.00, 122.93, 121.16, 116.99, 111.43, 105.76 (2 C, ArC-2′,6′), 61.01, 56.38 (2 C, OCH_3_-3′,5′), 55.71. HRMS calcd for C_23_H_23_N_2_O_4_ [M + H]^+^ 391.1658, found 391.1658.

##### 6-(3-methoxyphenyl)-3–(3,4,5-trimethoxyphenyl)imidazo[1,2-a]pyridine (8f)

Yield: 76%; M.p. 109.6–111.3 °C; ^1^H NMR (500 MHz, CDCl_3_) *δ* 8.47 (s, 1H, imidazole-H), 7.75-7.71 (m, 1H, ArH), 7.57 (d, *J* = 1.3 Hz, 1H, ArH), 7.45-7.44 (m, 1H, ArH), 7.38 (d, *J* = 8.0 Hz, 1H, ArH), 7.10 (d, *J* = 8.0 Hz, 1H, ArH), 7.05 (s, 1H, ArH), 6.94 (dd, *J* = 8.3, 2.1 Hz, 1H, ArH), 6.77 (s, 2H, ArH-2′,6′), 3.94 (s, 3H, OCH_3_-4′), 3.91 (s, 6H, OCH_3_-3′,5′), 3.86 (s, 3H, OCH_3_-3″); ^13^C NMR (126 MHz, CDCl_3_) *δ* 160.17, 154.01 (2 C, ArC-3′,5′), 144.83, 138.75, 132.92, 132.09, 131.80, 130.88, 130.28, 129.99, 129.71, 128.82, 125.38, 120.86, 119.33 (2 C, ArC-2′,6′), 113.18, 105.83, 61.00, 56.39 (2 C, OCH_3_-3′,5′), 55.37. HRMS calcd for C_23_H_23_N_2_O_4_ [M + H]^+^ 391.1658, found 391.1658.

##### 6-(4-methoxyphenyl)-3–(3,4,5-trimethoxyphenyl)imidazo[1,2-a]pyridine (8 g)

Yield: 92%; M.p. 129.2–130.2 °C; ^1^H NMR (500 MHz, CDCl_3_) *δ* 8.42 (s, 1H, imidazole-H), 7.66 (d, *J* = 7.0 Hz, 2H, ArH-2″,6″), 7.55 (td, *J* = 7.3, 1.3 Hz, 2H, ArH), 7.48 (d, *J* = 2.9 Hz, 1H, ArH), 6.99 (d, *J* = 8.7 Hz, 2H, ArH-3″,5″), 6.78 (s, 2H, ArH-2′,6′), 3.94 (s, 3H, OCH_3_-4′), 3.91 (s, 6H, OCH_3_-3′,5′), 3.85 (s, 3H, OCH_3_-4″); ^13^C NMR (126 MHz, CDCl_3_) *δ* 159.59, 153.96 (2 C, ArC-3′,5′), 145.16, 138.25, 133.03, 132.71, 129.90, 128.02 (2 C, ArC-2″,6″), 126.88, 125.11, 124.76, 120.02, 117.99, 114.61 (2 C, ArC-3″,5″), 105.68 (2 C, ArC-2′,6′), 61.04, 56.39 (2 C, OCH_3_-3′,5′), 55.42. HRMS calcd for C_23_H_23_N_2_O_4_ [M + H]^+^ 391.1658, found 391.1657.

##### 6-(4-ethoxyphenyl)-3–(3,4,5-trimethoxyphenyl)imidazo[1,2-a]pyridine (8h)

Yield: 83%; M.p. 58.3-59.6 °C; ^1^H NMR (500 MHz, CDCl_3_) *δ* 8.41 (s, 1H, imidazole-H), 7.67-7.65 (m, 2H, ArH-2″,6″), 7.57-7.53 (m, 2H, ArH), 7.43 (s, 1H, ArH), 6.98 (d, *J* = 8.7 Hz, 2H, ArH-3″,5″), 6.78 (s, 2H, ArH-2′,6′), 4.08 (q, *J* = 7.0 Hz, 2H, OCH_2_CH_3_), 3.94 (s, 3H, OCH_3_-4′), 3.91 (s, 6H, OCH_3_-3′,5′), 1.44 (t, *J* = 7.0 Hz, 3H, OCH_2_CH_3_); ^13^C NMR (126 MHz, CDCl_3_) *δ* 158.96, 153.95 (2 C, ArC-3′,5′), 145.16, 138.25, 133.04, 132.69, 129.71, 127.99 (2 C, ArC-2″,6″), 126.92, 125.12, 124.77, 119.99, 117.97, 115.14 (2 C, ArC-3″,5″), 105.69 (2 C, ArC-2′,6′), 63.63, 61.04, 56.39 (2 C, OCH_3_-3′,5′), 14.83. HRMS calcd for C_24_H_25_N_2_O_4_ [M + H]^+^ 405.1814, found 405.1816.

##### 4-(3–(3,4,5-trimethoxyphenyl)imidazo[1,2-a]pyridin-6-yl)phenol (8i)

Yield: 75%; M.p. 163.3–164.9 °C; ^1^H NMR (500 MHz, DMSO) *δ* 9.64 (s, 1H, OH), 8.56 (s, 1H, imidazole-H), 7.75 (s, 1H, ArH), 7.70 (d, *J* = 9.3 Hz, 1H, ArH), 7.57 (dd, *J* = 9.4, 1.7 Hz, 1H, ArH), 7.52 (d, *J* = 8.6 Hz, 2H, ArH-2″,6″), 7.00 (s, 2H, ArH-2′,6′), 6.87 (d, *J* = 8.6 Hz, 2H, ArH-3″,5″), 3.86 (s, 6H, OCH_3_-3′,5′), 3.74 (s, 3H, OCH_3_-4′); ^13^C NMR (126 MHz, DMSO) *δ* 157.82, 153.97 (2 C, ArC-3′,5′), 144.83, 137.82, 133.27, 128.36 (2 C, ArC-2″,6″), 127.90, 126.40, 126.10, 125.07, 124.79, 120.25, 117.79, 116.36 (2 C, ArC-3″,5″), 105.88 (2 C, ArC-2′,6′), 60.55, 56.62 (2 C, OCH_3_-3′,5′). HRMS calcd for C_22_H_21_N_2_O_4_ [M + H]^+^ 377.1501, found 377.1500.

##### 6-(4-fluorophenyl)-3-(3,4,5-trimethoxyphenyl)imidazo[1,2-a]pyridine (8j)

Yield: 69%; M.p. 168.5–169.3 °C; ^1^H NMR (500 MHz, CDCl_3_) *δ* 8.41 (s, 1H, imidazole-H), 7.77 (d, *J* = 8.5 Hz, 1H, ArH), 7.70 (s, 1H, ArH), 7.52-7.46 (m, 2H, ArH-2″,6″), 7.43 (d, *J* = 10.4 Hz, 1H, ArH), 7.16 (t, *J* = 8.6 Hz, 2H, ArH-3″,5″), 6.77 (s, 2H, ArH-2′,6′), 3.94 (s, 3H, OCH_3_-4′), 3.91 (s, 6H, OCH_3_-3′,5′); ^13^C NMR (126 MHz, CDCl_3_) *δ* 162.74, 154.02 (2 C, ArC-3′,5′), 145.10, 138.45, 133.55, 132.71, 129.29, 128.62 (2 C, ArC-2″,6″), 126.22, 125.09, 124.45, 120.58, 118.15, 116.17 (2 C, ArC-3″,5″), 105.82 (2 C, ArC-2′,6′), 61.03, 56.42 (2 C, OCH_3_-3′,5′). HRMS calcd for C_22_H_20_FN_2_O_3_ [M + H]^+^ 379.1458, found 379.1453.

##### 6-(4-chlorophenyl)-3-(3,4,5-trimethoxyphenyl)imidazo[1,2-a]pyridine (8k)

Yield: 86%; M.p. 162.1-162.9 °C; ^1^H NMR (500 MHz, CDCl_3_) *δ* 8.44 (s, 1H, imidazole-H), 7.74 (d, *J* = 9.3 Hz, 1H, ArH), 7.65 (d, *J* = 1.4 Hz, 2H, ArH-2″,6″), 7.55 (d, *J* = 1.5 Hz, 1H, ArH), 7.46 (d, *J* = 3.0 Hz, 2H, ArH-3″,5″), 7.42 (dd, *J* = 4.1, 2.0 Hz, 1H, ArH), 6.77 (s, 2H, ArH-2′,6′), 3.94 (s, 3H, OCH_3_-4′), 3.91 (s, 6H, OCH_3_-3′,5′); ^13^C NMR (126 MHz, CDCl_3_) *δ* 154.02 (2 C, ArC-3′,5′), 145.22, 138.43, 135.99, 134.12, 133.06, 133.01, 129.37 (2 C, ArC-2″,6″), 128.17 (2 C, ArC-3″,5″), 126.06, 124.62, 124.50, 120.68, 118.33, 105.76 (2 C, ArC-2′,6′), 61.04, 56.42 (2 C, OCH_3_-3′,5′). HRMS calcd for C_22_H_20_ClN_2_O_3_ [M + H]^+^ 395.1162, found 395.1162.

##### 6-(4-nitrophenyl)-3-(3,4,5-trimethoxyphenyl)imidazo[1,2-a]pyridine (8 l)

Yield: 73%; M.p. 220.0–221.6 °C; ^1^H NMR (500 MHz, CDCl_3_) *δ* 8.54 (s, 1H, imidazole-H), 8.33 (d, *J* = 8.8 Hz, 2H, ArH-3″,5″), 7.81 (d, *J* = 9.3 Hz, 1H, ArH), 7.79-7.59 (m, 3H, ArH-2″,6″ and ArH), 7.49 (dd, *J* = 9.4, 1.7 Hz, 1H, ArH), 6.77 (s, 2H, ArH-2′,6′), 3.95 (s, 3H, OCH_3_-4′), 3.91 (s, 6H, OCH_3_-3′,5′); ^13^C NMR (126 MHz, CDCl_3_) *δ* 154.12 (2 C, ArC-3′,5′), 147.45, 145.14, 143.93, 138.68, 138.53, 133.28, 127.59 (2 C, ArC-2″,6″), 126.60, 125.01, 124.52 (2 C, ArC-3″,5″), 124.07, 121.72, 118.74, 105.90 (2 C, ArC-2′,6′), 61.05, 56.46 (2 C, OCH_3_-3′,5′). HRMS calcd for C_22_H_20_N_3_O_5_ [M + H]^+^ 406.1403, found 406.1404.

##### 4-(3-(3,4,5-trimethoxyphenyl)imidazo[1,2-a]pyridin-6-yl)benzonitrile (8 m)

Yield: 71%; M.p. 213.3–215.1 °C; ^1^H NMR (500 MHz, CDCl_3_) *δ* 8.50 (s, 1H, imidazole-H), 7.76 (dd, *J* = 19.9, 11.5 Hz, 4H, ArH-2″,3″,5″,6″), 7.65 (d, *J* = 8.4 Hz, 2H, ArH), 7.45 (dd, *J* = 9.4, 1.7 Hz, 1H, ArH), 6.77 (s, 2H, ArH-2′,6′), 3.94 (s, 3H, OCH_3_-4′), 3.91 (s, 6H, OCH_3_-3′,5′); ^13^C NMR (126 MHz, CDCl_3_) *δ* 154.09 (2 C, ArC-3′,5′), 145.21, 142.05, 138.63, 133.29, 132.99 (2 C, ArC-2″,6″), 127.50 (2 C, ArC-3″,5″), 126.53, 125.33, 124.18, 124.04, 121.48, 118.70, 118.50, 111.70, 105.90 (2 C, ArC-2′,6′), 61.04, 56.46 (2 C, OCH_3_-3′,5′). HRMS calcd for C_23_H_20_N_3_O_3_ [M + H]^+^ 386.1505, found 386.1499.

##### 6-(3,4-dimethylphenyl)-3–(3,4,5-trimethoxyphenyl)imidazo[1,2-a]pyridine (8n)

Yield: 62%; M.p. 70.0–71.3 °C; ^1^H NMR (500 MHz, CDCl_3_) *δ* 8.45 (s, 1H, imidazole-H), 7.72 (d, *J* = 9.5 Hz, 1H, ArH), 7.65 (d, *J* = 1.4 Hz, 1H, ArH), 7.45 (dd, *J* = 2.8, 1.1 Hz, 1H, ArH), 7.30–7.21 (m, 3H, ArH), 6.79 (s, 2H, ArH-2′,6′), 3.94 (s, 3H, OCH_3_-4′), 3.91 (s, 6H, OCH_3_-3′,5′), 2.33 (s, 3H, ArCH_3_), 2.31 (s, 3H, ArCH_3_); ^13^C NMR (126 MHz, CDCl_3_) *δ* 153.96 (2 C, ArC-3′,5′), 145.25, 138.25, 137.46, 136.57, 135.01, 133.05, 132.70, 130.42, 128.17, 127.24, 125.23, 124.77, 124.25, 120.34, 117.94, 105.67 (2 C, ArC-2′,6′), 61.04, 56.38 (2 C, OCH_3_-3′,5′), 19.98, 19.47. HRMS calcd for C_24_H_25_N_2_O_3_ [M + H]^+^ 389.1865, found 389.1866.

##### 2-methoxy-5-(3-(3,4,5-trimethoxyphenyl)imidazo[1,2-a]pyridin-6-yl)phenol (8o)

Yield: 56%; M.p. 218.6–219.7 °C; ^1^H NMR (500 MHz, CDCl_3_) *δ* 8.41 (s, 1H, imidazole-H), 7.74 (d, *J* = 9.3 Hz, 1H, ArH), 7.68 (s, 1H, ArH), 7.46 (d, *J* = 9.3 Hz, 1H, ArH), 7.11 (d, *J* = 2.1 Hz, 1H, ArH-2″), 7.02 (dd, *J* = 8.3, 2.2 Hz, 1H, ArH-6″), 6.94 (d, *J* = 8.3 Hz, 1H, ArH-5″), 6.77 (s, 2H, ArH-2′,6′), 3.94 (s, 6H, OCH_3_-3′,5′), 3.91 (s, 6H, OCH_3_-4′ and OCH_3_-2″); ^13^C NMR (126 MHz, CDCl_3_) *δ* 153.95 (2 C, ArC-3′,5′), 146.69, 146.21, 144.85, 138.38, 132.05, 130.71, 127.07, 126.08, 125.41, 124.40, 120.20, 118.54, 117.69, 113.16, 111.18, 105.77 (2 C, ArC-2′,6′), 61.00, 56.37 (2 C, OCH_3_-3′,5′), 56.08. HRMS calcd for C_23_H_23_N_2_O_5_ [M + H]^+^ 407.1607, found 407.1604.

##### 6-(3,4-dimethoxyphenyl)-3-(3,4,5-trimethoxyphenyl)imidazo[1,2-a]pyridine (8p)

Yield: 75%; M.p. 67.3–68.9 °C; ^1^H NMR (500 MHz, CDCl_3_) *δ* 8.44 (s, 1H, imidazole-H), 7.75–7.67 (m, 2H, ArH), 7.45 (dd, *J* = 9.3, 1.7 Hz, 1H, ArH), 7.08 (dd, *J* = 8.3, 2.1 Hz, 1H, ArH-6″), 7.02 (d, *J* = 2.1 Hz, 1H, ArH-2″), 6.96 (d, *J* = 8.3 Hz, 1H, ArH-5″), 6.79 (s, 2H, ArH-2′,6′), 3.94 (s, 3H, OCH_3_-4′), 3.94 (s, 3H, OCH_3_-3″), 3.93 (s, 3H, OCH_3_-4″), 3.92 (s, 6H, OCH_3_-3′,5′); ^13^C NMR (126 MHz, CDCl_3_) *δ* 153.97 (2 C, ArC-3′,5′), 149.46, 149.13, 145.21, 138.26, 132.78, 130.40, 127.16, 126.06, 125.18, 124.74, 120.19, 119.38, 118.00, 111.73, 110.29, 105.61 (2 C, ArC-2′,6′), 61.04, 56.37 (2 C, OCH_3_-3′,5′), 56.06, 56.05. HRMS calcd for C_24_H_25_N_2_O_5_ [M + H]^+^ 421.1763, found 421.1765.

##### 6-(Naphthalen-2-yl)-3–(3,4,5-trimethoxyphenyl)imidazo[1,2-a]pyridine (8q)

Yield: 70%; M.p. 148.9–151.3 °C; ^1^H NMR (500 MHz, CDCl_3_) *δ* 8.60 (s, 1H, imidazole-H), 7.99 (d, *J* = 1.3 Hz, 1H, ArH-1″), 7.94 (d, *J* = 8.5 Hz, 1H, ArH), 7.91-7.86 (m, 2H, ArH), 7.79 (d, *J* = 9.3 Hz, 1H, ArH), 7.71 (d, *J* = 8.3 Hz, 2H, ArH), 7.62 (dd, *J* = 9.3, 1.7 Hz, 1H, ArH), 7.53–7.50 (m, 1H, ArH), 7.44 (dd, *J* = 2.8, 1.1 Hz, 1H, ArH), 6.81 (s, 2H, ArH-2′,6′), 3.94 (s, 3H, OCH_3_-4′), 3.92 (s, 6H, OCH_3_-3′,5′); ^13^C NMR (126 MHz, CDCl_3_) *δ* 154.02 (2 C, ArC-3′,5′), 144.56, 138.34, 134.75, 133.60, 133.05, 132.89, 132.78, 129.03, 128.09, 127.75, 127.13, 126.78, 126.45, 125.82, 125.17, 124.84, 124.67, 121.00, 118.22, 105.70 (2 C, ArC-2′,6′), 61.05, 56.40 (2 C, OCH_3_-3′,5′). HRMS calcd for C_26_H_23_N_2_O_3_ [M + H]^+^ 411.1709, found 411.1708.

##### 6-(Thiophen-3-yl)-3–(3,4,5-trimethoxyphenyl)imidazo[1,2-a]pyridine (8r)

Yield: 90%; M.p. 135.5–136.6 °C; ^1^H NMR (500 MHz, CDCl_3_) *δ* 8.51 (s, 1H, imidazole-H), 7.71 (d, *J* = 9.6 Hz, 1H, ArH), 7.66 (d, *J* = 7.0 Hz, 1H, thiophene-H), 7.48 (dd, *J* = 9.3, 1.6 Hz, 2H, ArH), 7.43 (s, 1H, thiophene-H), 7.30 (dd, *J* = 4.4, 2.0 Hz, 1H, thiophene-H), 6.78 (s, 2H, ArH-2′,6′), 3.94 (s, 3H, OCH_3_-4′), 3.92 (s, 6H, OCH_3_-3′,5′); ^13^C NMR (126 MHz, CDCl_3_) *δ* 154.00 (2 C, ArC-3′,5′), 145.16, 138.29, 132.76, 132.06, 128.46, 127.20, 126.15, 125.67, 124.58, 122.21, 120.86, 120.02, 118.19, 105.69 (2 C, ArC-2′,6′), 61.04, 56.41 (2 C, OCH_3_-3′,5′). HRMS calcd for C_20_H_19_N_2_O_3_S [M + H]^+^ 367.1116, found 367.1117.

##### 6-(Pyridin-3-yl)-3–(3,4,5-trimethoxyphenyl)imidazo[1,2-a]pyridine (8s)

Yield: 88%; M.p. 170.3–171.5 °C; ^1^H NMR (500 MHz, CDCl_3_) *δ* 8.82 (d, *J* = 2.1 Hz, 1H, pyridine-H-2″), 8.64 (dd, *J* = 4.8, 1.4 Hz, 1H, pyridine-H-6″), 8.49 (s, 1H, imidazole-H), 7.88-7.82 (m, 1H, pyridine-H-4″), 7.80 (d, *J* = 9.3 Hz, 1H, ArH), 7.72 (s, 1H, ArH), 7.47-7.38 (m, 2H, pyridine-H-5″ and ArH), 6.78 (s, 2H, ArH-2′,6′), 3.94 (s, 3H, OCH_3_-4′), 3.92 (s, 6H, OCH_3_-3′,5′); ^13^C NMR (126 MHz, CDCl_3_) *δ* 154.06 (2 C, ArC-3′,5′), 149.19, 147.97, 145.16, 138.54, 134.33, 133.31, 133.06, 126.43, 124.35, 124.28, 123.93, 123.85, 121.05, 118.69, 105.78 (2 C, ArC-2′,6′), 61.04, 56.42 (2 C, OCH_3_-3′,5′). HRMS calcd for C_21_H_20_N_3_O_3_ [M + H]^+^ 362.1505, found 362.1501.

##### 6-(Pyridin-4-yl)-3–(3,4,5-trimethoxyphenyl)imidazo[1,2-a]pyridine (8t)

Yield: 64%; M.p. 125.2–127.1 °C; ^1^H NMR (500 MHz, CDCl_3_) *δ* 8.70 (d, *J* = 5.5 Hz, 2H, pyridine-H-2″,6″), 8.57 (s, 1H, imidazole-H), 7.80 (d, *J* = 9.4 Hz, 1H, ArH), 7.73 (s, 1H, ArH), 7.49 (dd, *J* = 15.3, 3.8 Hz, 3H, ArH and pyridine-H-3″,5″), 6.77 (s, 2H, ArH-2′,6′), 3.95 (s, 3H, OCH_3_-4′), 3.92 (s, 6H, OCH_3_-3′,5′); ^13^C NMR (126 MHz, CDCl_3_) *δ* 154.10 (2 C, ArC-3′,5′), 150.64, 145.41, 144.93, 138.61, 133.33, 129.29, 128.88, 126.56, 124.27, 124.16, 123.64, 121.54, 121.23, 118.76, 105.83 (2 C, ArC-2′,6′), 61.04, 56.45 (2 C, OCH_3_-3′,5′). HRMS calcd for C_21_H_20_N_3_O_3_ [M + H]^+^ 362.1505, found 362.1498.

##### 6-(1H-indol-4-yl)-3–(3,4,5-trimethoxyphenyl)imidazo[1,2-a]pyridine (8 u)

Yield: 70%; M.p. 258.2–259.6 °C; ^1^H NMR (500 MHz, CDCl_3_) *δ* 8.77 (s, 1H, indole-NH), 8.61 (s, 1H, imidazole-H), 7.79 (d, *J* = 7.6 Hz, 1H, ArH), 7.73 (s, 1H, ArH), 7.62 (d, *J* = 8.8 Hz, 1H, indole-H), 7.45 (d, *J* = 8.1 Hz, 1H, indole-H), 7.31–7.29 (m, 1H, indole-H), 7.27 (d, *J* = 7.9 Hz, 1H, ArH), 7.16 (d, *J* = 7.0 Hz, 1H, ArH), 6.81 (s, 2H, ArH-2′,6′), 6.68 (s, 1H, indole-H-3), 3.91 (s, 9H, OCH_3_-3′,4′,5′); ^13^C NMR (126 MHz, CDCl_3_) *δ* 153.92 (2 C, ArC-3′,5′), 145.30, 138.21, 136.32, 132.49, 130.14, 128.63, 127.11, 126.88, 126.16, 125.05, 124.73, 122.41, 122.02, 119.71, 117.63, 111.09, 105.63 (2 C, ArC-2′,6′), 101.31, 61.01, 56.34 (2 C, OCH_3_-3′,5′). HRMS calcd for C_24_H_22_N_3_O_3_ [M + H]^+^ 400.1661, found 400.1663.

### Biological evaluation

#### In vitro anti-proliferative activity

The antiproliferative activities of the synthesised compounds and the reference agent **CA-4** were evaluated against a panel of human cancer cell lines using the standard MTT assay^42^. Cells were seeded into 96-well plates (Corning, USA) at a density of 5 × 10³ cells per well and allowed to adhere for 24 h. Subsequently, cells were treated with either the test compounds (0–100 μM, dissolved in 0.1% DMSO, Rhawn Chemicals, R012315, 99.7%) or vehicle control (0.1% DMSO) for 72 h. Following incubation, the medium was replaced with MTT reagent (Roche, 11465007001) and further incubated for 4 h. The resulting formazan crystals were solubilised in DMSO (150 μL/well, Rhawn Chemicals, R012315, 99.7%) under gentle shaking for 10 min. Absorbance was measured at 492 nm using a microplate reader (BioTek, Synergy H1). IC_50_ values were determined by nonlinear regression analysis using SPSS software (Version 26.0, IBM, USA). All cell lines, including HeLa (NCACC, SCSP-504), HCT116 (NCACC, SCSP-5076), MCF-7 (NCACC, SCSP-531), and HUVECs (NCACC, PSC-01) were obtained from the Cell Bank of the Chinese Academy of Sciences (Shanghai, China) and maintained under recommended culture conditions.

#### Effect on tubulin polymerisation

The effects of the target compounds on microtubule dynamics were assessed using a commercial tubulin polymerisation assay kit (BK001, Cytoskeleton Inc., Denver, CO, USA) according to the manufacturer’s protocol^44^. Tubulin protein was reconstituted in ice-cold G-PEM buffer (80 mM PIPES, 1 mM GTP, 0.5 mM EGTA, 2 mM MgCl_2_, 15% glycerol, pH 6.9). Compound **8o** (3 μM), PTX (5 μM, positive control for microtubule stabilisation, Sigma-Aldrich, MFCD00869953), and **CA-4** (3 μM, positive control for microtubule destabilisation, Sigma-Aldrich, MFCD03453309) were pre-incubated with the tubulin solution prior to initiation of polymerisation. The reaction mixtures were transferred to a pre-warmed 96-well plate and maintained at 37 °C. Polymerisation kinetics were monitored fluorometrically every 60 s for 60 min (excitation 360 nm, emission 420 nm) using an Infinite^®^ F500 microplate reader (Tecan, Switzerland). The fluorescence intensity over time was analysed to determine the rate and extent of tubulin polymerisation in the presence of each test compound.

#### Analysis of immunofluorescence staining

Immunofluorescence analysis was performed according to previously published protocols^43^. HeLa cells were seeded into 24-well culture plates (Corning, USA) and allowed to adhere overnight. Following treatment with vehicle, **8o**, or **CA-4** for 24 h, cells were permeabilized with 0.2% Triton X-100 (Sigma-Aldrich, MFCD00128254) for 10 min at room temperature, then fixed with 4% paraformaldehyde (Beyotime, P0099) for 15 min. After washing with PBS (Beyotime, C0221B), non-specific binding sites were blocked by incubating with 5% bovine serum albumin (BSA; Beyotime, ST023) in PBS for 1 h at room temperature. Cells were subsequently incubated with a monoclonal anti-*α*-tubulin antibody (Beyotime, AF2827; 1:100 dilution in PBS containing 2% BSA) overnight at 4 °C. After three washes with PBS, samples were exposed to FITC-conjugated goat anti-mouse IgG secondary antibody (Abclonal, GTX300120; 1:100 in PBS with 2% BSA) for 2 h at 37 °C in the dark. Nuclei were counterstained with DAPI (Beyotime, C1006; 1 μg/mL) for 5 min. Following final washes, the immunofluorescent signals were imaged using a confocal laser scanning microscope (Zeiss LSM 880, Germany).

#### Cell cycle analysis

Cell cycle distribution was determined by propidium iodide (PI)-based flow cytometry following established methodology^44^. HeLa cells were treated with compound **8o** or vehicle control (0.05% DMSO) for 24 h. After treatment, cells were harvested by trypsinization, washed with cold PBS, and fixed in 70% ice-cold ethanol (Sigma-Aldrich, 02851) for at least 2 h at −20 °C. Following fixation, cells were washed twice with PBS and resuspended in PI/RNase staining buffer (Beyotime, C1734) containing 50 μg/mL propidium iodide (Beyotime, ST1569). Samples were incubated for 30 min at room temperature in the dark. DNA content was analysed using a BD FACSVerse flow cytometer (BD Biosciences, USA), and cell cycle profiles were quantified with FlowJo software (Version 10, TreeStar, USA). The percentages of cells in G0/G1, S, and G2/M phases were determined using built-in cell cycle modelling algorithms.

#### Induction of cell apoptosis

Apoptosis induced by compound **8o** was quantified using Annexin V-FITC/propidium iodide (PI) dual staining coupled with flow cytometry, as described previously^44^. HeLa cells were treated with **8o** at concentrations of 50, 100, and 150 nM for 48 h. After treatment, cells were harvested, washed twice with cold PBS, and resuspended in 500 μL of 1× Annexin V binding buffer. Subsequently, 5 μL of Annexin V-FITC (Beyotime, C1062S) and 5 μL of propidium iodide (Beyotime, ST1569) were added to each sample, followed by gentle mixing and incubation for 15 min in the dark at room temperature. Apoptotic analysis was performed immediately using a BD FACScan flow cytometer (BD Biosciences, USA), and data were processed with FlowJo software (Version 10, TreeStar, USA). The percentages of early apoptotic (Annexin V-FITC^+^/PI^-^), late apoptotic (Annexin V-FITC^+^/PI^+^), and necrotic (Annexin V-FITC^-^/PI^+^) cells were determined using quadrant analysis.

### Molecular docking analysis

Molecular docking studies were performed using the Schrödinger Suite 2022–1. The three-dimensional structure of tubulin complexed with a colchicine-site ligand (PDB ID: 5LYJ) was retrieved from the Protein Data Bank. Prior to docking, the protein structure was prepared using the Protein Preparation Wizard within Maestro. This involved assigning bond orders, adding hydrogen atoms, filling missing side chains using Prime, and optimising the hydrogen-bonding network. The structure was subsequently minimised with the OPLS4 force field to relieve steric clashes.

All ligand structures were initially drawn in ChemBioDraw Ultra (Version 20.0) and converted to three-dimensional format using LigPrep. Ligand ionisation states were generated at pH 7.0 ± 2.0 with Epik, and low-energy conformers were retained.

The colchicine-binding site was defined as the centroid of the co-crystallized ligand. A receptor grid box of dimensions 15 Å × 15 Å × 15 Å was generated to encompass the binding pocket. Docking simulations were conducted with the Glide module in standard precision (SP) mode. Post-docking analysis, including visualisation of hydrogen bonds, hydrophobic contacts, and binding poses, was performed using PyMOL (Version 2.5).

## Data Availability

The datasets used and/or analysed during the current study are available from the corresponding author upon reasonable request.
